# I like what you are saying, but only if i feel safe: Psychological safety moderates the relationship between voice and perceived contribution to healthcare team effectiveness

**DOI:** 10.3389/fpsyg.2023.1129359

**Published:** 2023-04-17

**Authors:** Mona Weiss, Elizabeth W. Morrison, Demian Szyld

**Affiliations:** ^1^School of Business and Economics, Department of Management, Freie Universität Berlin, Berlin, Germany; ^2^Stern School of Business and Economics, Department of Management, New York University, New York, NY, United States; ^3^School of Medicine, Boston University, Boston, MA, United States

**Keywords:** psychological safety, voice, teamwork, healthcare, nurses, hierarchy

## Abstract

**Introduction:**

Are nurses who voice work-related concerns viewed as positive contributors to a team? We propose that the extent to which healthcare professionals consider voice by nurses as helpful for the team depends on how psychologically safe they feel. Specifically, we hypothesized that psychological safety moderates the relationship between voice of a lower ranking team member (i.e., a nurse) and perceived contribution by others, such that voice is more likely to be seen as valuable for team decision-making when psychological safety is high but not when it is low.

**Methods:**

We tested our hypotheses with a randomized between-subjects experiment using a sample of emergency medicine nurses and physicians. Participants evaluated a nurse who either did or did not speak up with alternative suggestions during emergency patient treatment.

**Results:**

Results confirmed our hypotheses: At higher levels of psychological safety the nurse’s voice was considered as more helpful than withholding of voice for team decision-making. This was not the case at lower levels of psychological safety. This effect was stable when including important control variables (i.e., hierarchical position, work experience, gender).

**Discussion:**

Our results shed light on how evaluations of voice are contingent on perceptions of a psychologically safe team context.

## Introduction

Successful teamwork depends on effective and efficient sharing of information among individuals (Mesmer-Magnus and DeChurch, 2009). Healthcare teams in particular need to discuss treatment options and potential hazards to ensure patient safety (e.g., Castelao et al., 2013; Hautz et al., 2015). Yet, research suggests that individuals are often reluctant to speak up with alternative suggestions or concerns that challenge the status quo (i.e., voice) because they fear backlash from co-workers and superiors (Morrison and Milliken, 2000). In healthcare, these fears are especially prevalent as nurse’s report that they are often hesitant to voice suggestions or concerns to physicians (Russo et al., 2015).

Existing research on consequences of voice has shown that speaking up has the potential to result in positive evaluations from superiors but that these are contingent on factors such as demographic characteristics (e.g., gender, ethnicity) of the voicer (Howell et al., 2015), or on how, what, and when they voice (Whiting et al., 2012; Maynes and Podsakoff, 2014; Chamberlin et al., 2017). Further, a qualitative study by Burris et al. (2017) suggested that in healthcare contexts, the content of the voiced message and the ease of enacting the recommended change affected the extent to which manager’s value voice behavior. At this point, however, we know relatively little about how voice is evaluated in healthcare teams that come together on the spot to engage in rapid decision-making and that are characterized by an entrenched professional hierarchy (e.g., Cott, 1997; Nembhard and Edmondson, 2006; Uitdewilligen and Waller, 2018). Thus, to advance current knowledge on receptivity to voice, it is crucial to better understand the conditions under which the voicing of suggestions and concerns by a lower ranking team member (i.e., a nurse) is viewed as helpful by other team members.

In this research, we build on previous findings that show that constructive voice can get individuals more recognition and appreciation from others, as they are seen as contributing positively to a team (e.g., Weiss and Morrison, 2019). We argue that healthcare team members consider it as more helpful for the team when a lower-ranking team member speaks up than remains silent with his/her concerns. Yet, we posit that this effect is contingent on team members’ psychological safety. Drawing from a social-cognitive perspective, we argue that when people perceive and evaluate other people’s behavior, they are strongly influenced by their experiences of and resulting beliefs about the social context (e.g., Fiske, 1993). Psychological safety refers to the perception of whether the organizational or team context is safe for interpersonal risk-taking (Edmondson, 1999; Edmondson and Lei, 2014) and is also a crucial antecedent of engaging in voice (Detert and Burris, 2007). Feeling psychologically safe (i.e., being able to bring up problems and tough issues) is especially important in healthcare settings where individuals are strongly attuned to hierarchical norms within the team and expectations of superiors (Nembhard and Edmondson, 2006).

We argue that psychological safety not only determines whether people voice themselves, but also affects the extent to which they perceive voice by others as helpful for the team. We argue that only when people *themselves* feel safe to ask questions and point out problems, will they also appreciate it when *others*—especially those who hold a lower formal rank and are usually expected to agree with the suggestions of higher-ranking individuals—take a risk and voice their opinion. We tested this prediction in a medical emergency team context, where speaking up with alternative suggestions or concerns regarding patient treatment is crucial for team decision-making but is particularly risky for team members from a subordinate profession (i.e., nurses) who hold relatively lower status and power (Cott, 1997; Magee and Galinsky, 2008).

With this study, we contribute to and extend the growing body of work that focuses on the consequences of voice for the individual employee (e.g., Burris, 2012; Whiting et al., 2012; Weiss and Morrison, 2019, see also Morrison, 2023 for an overview). We point to psychological safety as an important social-contextual factor affecting evaluations of voice in functionally and hierarchically diverse *ad-hoc* teams that operate in a high-risk context. Thus, psychological safety is not only a crucial antecedent of voice (Detert and Burris, 2007) but also poses a boundary conditions to evaluations of voice.

We also contribute to and extend the literature on healthcare team functioning by pointing out conditions under which healthcare team members appreciate voice behavior by nurses which can have important implications for the performance of these teams (e.g., Schmutz and Manser, 2013; Uitdewilligen and Waller, 2018). More broadly, our work complements research on functionally diverse teams that has suggested psychological safety as a moderator in the relationship between minority dissent or conflict and team performance (e.g., Bradley et al., 2012; Nijstad et al., 2014; see also Jetten and Hornsey, 2014). Over and above that, our findings suggest that psychological safety also affects whether individuals are able to value dissenting and challenging views of team members who have a lower professional rank and lower status on the team.

### Employee voice and perceived contribution to the team

In line with Morrison (2014), we define voice as “discretionary communication by an employee of ideas, suggestions, concerns, information about problems, or opinions about work-related issues […] with the intent to bring about improvement or change” (p. 174). There is evidence across several different industries that employee voice can have direct effects on organizational, unit, and team performance (Detert et al., 2013) and is particularly relevant for healthcare team effectiveness (Weiss et al., 2014). Yet, at this point, no research has systematically investigated how individuals who speak up in healthcare teams are evaluated by others. In this research, we are especially interested in how nurses’ voice is judged, because nurses are formally subordinate to physicians and as a result of this hierarchical difference, they may be less expected to challenge their decisions (e.g., Cott, 1997; Magee and Galinsky, 2008). Specifically, we aim to examine how voice relates to perceived contribution to team effectiveness, defined as the extent to which a behavior is considered as helpful for the team’s performance (Waller et al., 2011; Steinmetz et al., 2016). Such evaluations are particularly important during team decision-making and affect how team members elaborate and process divergent information (Jetten and Hornsey, 2014).

Two recent studies showed that voice can earn individuals higher status in the eyes of others (McClean et al., 2018; Weiss and Morrison, 2019). For example, Weiss and Morrison (2019) showed that constructive voice can lead to evaluations of higher agency, communion, and status as compared to silence within product development teams. These positive effects also appeared when the voiced suggestions came from a team member with a relatively lower rank. Relatedly, McClean et al. (2018) showed that team members who voiced promotively (i.e., voicing suggestions for improvement) were seen as higher status and were consequently more likely to be selected as leaders.

Based on these previous findings, we submit that voice should generally be considered as positive within healthcare teams as well. When team members with a lower occupational rank (i.e., nurses) provide alternative viewpoints and make suggestions regarding treatment options, this should signal to others that they are motivated to contribute to the team and that they have the patient’s best interest in mind (Weiss and Morrison, 2019). By voicing their concerns, they stimulate divergent thinking in teams by contributing their expertise and judgments (Jetten and Hornsey, 2014). As a result, they are likely to be seen as more competent and helpful team members than those who do not voice their concerns and suggestions. Thus, we hypothesize:

Hypothesis 1: Nurses’ voice is more likely to be seen as a positive contribution to team effectiveness than nurses’ withholding of voice.

### The moderating role of psychological safety in healthcare teams

Even though the literature has shown that voice has the potential to result in positive consequences for the voicing employee, studies point to a number of contingencies. For example, research by Burris (2012) showed that managers feel more threatened and perceive employees as more disloyal when they engage in challenging rather than supportive voice. Further specifying these effects, a study by Whiting et al. (2012) found that voicers who are perceived as more trustworthy, who speak up early rather than late during a discussion, and who frame their message constructively are seen as better performers. Moreover, Lam et al. (2019) found that individuals who spoke up in a direct rather than an indirect manner were more likely to be endorsed by their managers such that managers perceived their comments as valuable and helpful. Another study showed that employees get more credit for voicing when they have higher ascribed status, such as being from the majority ethnicity or working full-time (Howell et al., 2015). In sum, the existing research suggests that factors pertaining to the person who is voicing, or to the nature of the voiced message, affect how others react.

The current study points to an additional contingency factor, rooted in the social context. Our theoretical reasoning is informed by a social-cognitive perspective, which argues that individuals make sense of their social context based on processing social cues in the environment (Fiske, 1993). In other words, employees socially construe their beliefs, perceptions, and attitudes about the “right” way to think, feel, and behave within their organization. We argue that such social-cognitive processing should be especially relevant in healthcare teams because this context places emphasis on norms and codes of conduct defined by occupational function and hierarchical ordering (Cott, 1997).

One important social cue within work contexts is psychological safety, defined as the extent to which employees perceive their team or work unit as safe for interpersonal risk-taking (Edmondson, 1999). Psychological safety builds on the notion that employees hold tacit beliefs about interpersonal interactions at work, which can affect whether employees seek feedback, talk about errors or work-related concerns, and share information vertically and horizontally (Argyris, 1993). When employees feel that they can talk about ideas without being judged or disrespected, when they are given autonomy and freedom to engage with their work and develop new approaches, they feel psychologically safe at work. Kahn (1990) concluded that “psychological safety was experienced as feeling safe to show and employ one’s self without fear of negative consequences to self-image, status, and career” (Kahn, 1990, p. 708). Psychological safety has been shown to affect outcomes at multiple levels including employee engagement, team learning and performance, organizational performance, and organizational change processes (Edmondson, 1999; Baer and Frese, 2003; Cataldo et al., 2009; Edmondson et al., 2001). In the voice literature, psychological safety has often been conceived of as an important antecedent of speaking up (e.g., Detert and Burris, 2007).

Research has also demonstrated that psychological safety is of particular importance in healthcare settings. Studying an intensive care unit, Nembhard and Edmondson (2006) showed that unit-level psychological safety mediated the relationship between inclusive leadership and engagement in quality improvement efforts (i.e., being actively involved in efforts to improve work processes) suggesting psychological safety as a key mechanism for speaking up. One reason is that healthcare teams are hierarchically structured with clear professional boundaries and authority gradients between nurses and physicians (e.g., Cott, 1997). This creates not only a functional differentiation between nurses and physicians but also certain role expectations regarding power, status, and conformity (Apker et al., 2005). For example, it is commonly expected that physicians make important treatment decisions and that nurse’s take on an assisting function (Cott, 1997).

Another reason that psychological safety is particularly important in healthcare teams is the fact that they are *ad-hoc* teams and required to “team up” for a specific shift or a sudden unexpected emergency (e.g., Sundstrom et al., 1990). As such, they have also been described as “fluid teams” as they are constantly reconstituted and often have little shared history (Avgerinos et al., 2020). For example, in emergency care, teams are composed for a specific shift only and may even be recomposed during a specific teamwork episode (e.g., more nurses or physicians joining an EM team for a resuscitation; Hunziker et al., 2011). These circumstances place particular demands on healthcare teams, and, thus, previous research has emphasized the importance of socio-contextual factors such as familiarity or leadership (Avgerinos et al., 2020; Krenz et al., 2020; Akşin et al., 2021).

Psychological safety was originally proposed as a climate factor—or a shared perception—within teams or units (Edmondson, 1999). More recently, however, researchers have suggested that psychological safety should be conceptualized as a multi-level construct that can exist at the interpersonal, team, and unit level (Roussin et al., 2016). A recent study also reveals that within healthcare teams, psychological safety is shaped by multiple, accumulated teamwork episodes (e.g., O’Donovan et al., 2021). Thus, given the fluid nature of teams in healthcare and emergency care specifically, we conceptualize psychological safety at the level of the unit or department in which individuals are situated and work together in various and constantly changing team compositions. Even though we do not expect that perceptions of psychological safety will necessarily be shared within the context of *ad-hoc* teams, we believe that it will significantly impact the link between voice and perceived contribution to team effectiveness.

In more stable teams (i.e., those that remain intact over a longer period of time), psychological safety has been shown to be an important moderator in the context of team conflict and performance. For example, Bradley et al. (2012) investigated student project teams and found that team conflict was positively associated with team performance when teams perceived their psychological safety to be high, but not when they perceived it to be low. Moreover, conducting a study with top management teams, Nijstad et al. (2014) showed that psychological safety moderated the impact of minority dissent on team innovation such that dissent could only be transformed into innovative team outcomes when team members felt psychologically safe. This line of research shows that psychological safety can affect important team processes and outcomes and that it is especially important for teams dealing with dissent and conflict (Jetten and Hornsey, 2014).

Taken together, previous research suggests that psychological safety is often a prerequisite for engaging in voice and is especially important within teams and healthcare settings. Yet, it is unclear how psychological safety may affect interpersonal evaluations of voice—specifically when these evaluations are situated in healthcare teams that are marked by an entrenched social hierarchy.

Based on a social-cognitive approach (Fiske, 1993), we argue that individuals draw on their socially-informed cognitive schemas and belief systems to evaluate others’ voice behavior and one such schema is their psychological safety. We argue that when healthcare professionals feel psychologically safe, they will see more value in others who speak up in comparison to those who do not. Individuals who feel psychologically safe generally interpret their work context as a safe place for sharing alternative opinions and concerns (Edmondson and Lei, 2014). They feel that they can bring up tough issues at work and stand out from the group without being negatively judged (Edmondson, 1999). As a result of these perceptions and experiences, they have formed a positive schema about their work context in general (Fiske, 1993) which entails that they and others can freely voice opinions or concerns. Thus, these perceptions are likely to influence subsequent team interactions (O’Donovan et al., 2021) such that they will be more likely to perceive team members who speak up with suggestions as contributing positively to team decision-making, regardless of the person’s function or status. This is because they interpret their voice behavior as compatible with the perception that their work context allows for interpersonal risk-taking and as stimulating reflection and fostering team decision-making (Carmeli and Gittell, 2009; Edmondson, 1999; Bradley et al., 2012).

In contrast, when people feel psychologically unsafe at work, they should be less likely to view others’ voice as helpful for the team. Because they themselves feel unable to raise alternative suggestions or concerns, they may also use this schema to evaluate others’ behavior. When individuals feel psychologically unsafe, they should be less supportive of lower-ranking team members who propose alternative actions. This is because they hold the belief that alternative viewpoints are negatively judged, and that individuals who make mistakes will be punished (Edmondson, 1999). Individuals who feel psychologically unsafe may feel that it is not advisable to be different from others and stand out with one’s opinion, as they are highly concerned that such behavior results in negative interpersonal consequences (Edmondson, 1999; Bradley et al., 2012). Particularly if the person who voices has a lower hierarchical rank, they may see such behavior as a threat to the hierarchy and as crossing a line that they personally would not dare to cross (Nembhard and Edmondson, 2006). Consequently, they are less likely to view a lower-ranking team member who speaks up with concerns as helpful for the team. We thus hypothesize that the relationship between nurses’ voice and perceived contribution to the team is moderated by psychological safety:

Hypothesis 2: There is an interaction between nurses’ voice and psychological safety on perceived contribution to the team: At higher (but not at lower) levels of psychological safety, nurses are more likely to be judged as helpful for the team when they voice than when they do not voice their concerns.

## Materials and methods

### Participants

Participants were recruited from the Emergency Medicine (EM) department of a large hospital in the United States (Institutional Review Board approval number: i16-01193). We used the department’s email list to contact all of their physician and nursing staff (*N* = 250). Of these 250, 101 healthcare professionals participated in the study (overall response rate: 40.4%). Seventy-five participants were EM physicians and 26 were EM nurses. Fifty-three participants were female, and the average clinical work experience was 7.18 years (*SD* = 9.50). In comparison to the full population in this particular hospital department, our sample entailed more physicians (74 vs. 57) and fewer nurses (26 vs. 43%) and slightly fewer women (53 vs. 65%).

No incentive was given for participation, and all participants provided their informed consent to the use of their data.

### Materials

To systematically examine receptivity to nurses’ voice, we designed four clinical vignettes. Clinical vignettes are widely used in medical education and have also been used in previous studies on voice behavior in healthcare (Kobayashi et al., 2006). Our goal in developing these vignettes was to present a common but complex situation occurring during a patient assessment, where voicing an alternative suggestion can contribute to a more effective team decision in terms of higher quality of care and lowered risk for the patient.

All vignettes were identical with respect to the clinical case they described but differed with respect to whether a nurse spoke up with treatment-related concerns or remained silent with those concerns. Specifically, it was described that a 25-year old male patient was admitted to the Emergency Room (ER) with multiple injuries following a severe car accident. The case posed ambiguity as to how to proceed with the treatment (i.e., transferring patient immediately to radiology vs. applying further measures to stabilize the patient). The Glasgow Comma Scale (GCS) for this patient is 12 indicating mild to moderate traumatic brain injury. The Oxygen saturation is 90%, which in the context of rib injuries with suspected rib fractures could be due to lung contusions, pneumothorax (punctured lung), splinting/hypoventilation (when the lung is not well expanded due to pain), or from a combination of factors. Application of oxygen to raise the oxygen saturation to greater 92–94% would be standard. British Thoracic Society Guidelines recommend nasal cannula supplementation in this case (O’Driscoll et al., 2017). Intubation (placing a breathing tube) to ventilate and oxygenate would be indicated if the patient was declining in mental status, blood pressure or if oxygen saturation was not improving with supplemental oxygen. One often discussed reason for intubation is anticipating clinical course. One concern may be that traveling to the CT scanner and having to lie flat for a prolonged period of time would be safer if an intubation was performed in advance.

Participants were asked to adopt their regular professional role (nurse or physician) during the comprehension of the presented medical case. It was stated that a nurse on the team was concerned about the relatively low oxygen saturation of the patient and felt that it was necessary to intubate him immediately. This information was held constant across conditions, but we manipulated the nurse’s communication behavior. In the voice condition (*n* = 48), she spoke up with her concerns to the physicians and suggested intubating the patient before transferring him to the radiology department. In the no-voice condition (*n* = 53), she did not bring up her concerns or offer this alternative suggestion. We slightly adapted the wording of the vignette for nurses to match their perspective of evaluating a fellow nurse speaking up to a physician. Thus, for each of the two professional groups (nurses and physicians), we designed two versions of the clinical vignette (voice, no voice). Appendix A presents the full vignettes we used in the study.

### Procedure

The study was implemented as an online experiment, and EM nurses and EM physicians working in the study hospital were invited *via* email. We employed a randomized between-subjects design, such that each participant was assigned to one experimental condition only. After providing their informed consent, participants first provided information on their demographics including their gender, their age (assessed categorically to further ensure anonymity), and their professional function (nurse or physician). Next, participants were asked to report their perceived psychological safety in their department. After that, participants were randomly assigned to either the voice or the no-voice condition that matched their professional role on the team (i.e., either nurse or physician). After reading the vignette, participants completed a manipulation check question and were then asked to evaluate the nurse’s behavior in terms of its contribution to the team’s decision-making effectiveness. At the end of the study, participants received a comprehensive debriefing on the study purpose and the two different experimental conditions.

### Measures

#### Manipulation check

To ensure that our manipulation *via* the vignettes was effective, we used a manipulation check question (*Please indicate the extent to which you feel the nurse spoke up with alternative suggestions during the assessment*) with responses made on a scale from 1 (not at all) to 7 (very much).

#### Perceived contribution to team effectiveness

Based on established measures assessing perceived contribution in teams (e.g., Waller et al., 2011; Steinmetz et al., 2016), we asked participants to indicate the extent to which they agreed with the following item “This person’s behavior helped us make the right decision” using a scale from 1 (do not agree) to 7 (completely agree). Notably, this assessment presented a subjective perception of whether the respective behavior of the nurse contributed positively to the optimal team decision. Thus, either behavior, remaining silent or voicing an alternative treatment suggestion, could have been perceived as contributing positively to the team. Remaining silent with the suggestion to intubate could be seen as contributing to a more efficient team decision-making process as it allows for a faster further treatment (transferring to radiology without the need to intubate beforehand). Based on medical guidelines, however, oxygen levels below 90% are considered beginning desaturation which represents a major risk factor in ongoing emergency treatment and thus provide an indication for intubation (Dunford et al., 2003). Moreover, one major reason for intubation is anticipating clinical course. Considering the patient’s clinical status, one might worry that traveling to the CT scanner and having to lie flat for a prolonged period of time would be safer if an intubation was performed in advance. Thus, the decision on how to further treat the patient is not clearly apparent, but intubating the patient can be considered as the safer option and should certainly be openly discussed within the team to ensure the best possible outcome for the patient.

#### Psychological safety

We assessed psychological safety by using seven-item team psychological safety scale of Edmondson (1999) (Cronbach’s Alpha = 0.77). It is important to point out that we asked participants to report on their perceptions of psychological safety in their actual work department and not within the hypothetical scenario. Sample items were: “People in this department sometimes reject others for being different” or “If you make a mistake it is often held against you” using a scale from 1 (do not agree) to 7 (completely agree). We note that the intraclass correlation indicated poor agreement among the staff members from this particular EM department [ICC(1) = 0.28, *p* < 0.001], thus suggesting that there is variance in perceptions (Koo and Li, 2016).

#### Control variables

We assessed participants’ professional role (0 = nurse, 1 = physician) as a control variable, as it might affect evaluations of a member from one’s own or a different function. Moreover, in healthcare teams specifically, professional role and hierarchical level are intertwined such that physicians hold a higher hierarchical level than nurses which may further affect evaluations of voice. In addition, we controlled for participants’ gender (1 = male, 2 = female) and work experience (in years) as these have been noted as important in the context of voice (e.g., Howell et al., 2015).

## Results

Table 1 presents the means, standard deviations, and intercorrelations between all variables. Before testing our hypotheses, we examined the responses to our manipulation check question. The results confirmed that our manipulation of voice was successful, as participants in the voice condition reported a significantly higher extent of the nurse speaking up with alternative suggestions during the scenario (*M* = 5.79, *SD* = 1.20) than participants in the no-voice condition (*M* = 2.47, *SD* = 2.03), *t*(93) = −9.73, *p* < 0.001. We excluded eight participants who failed the manipulation check from further analyses^1^.

**Table 1:** Descriptive statistics and correlations.

Variable	*M*	*SD*	1	2	3	4	5
1. Professional role	0.76	0.43	—				
2. Work experience	7.10	9.52	0.00	—			
3. Gender	1.53	0.50	−0.33^*^^*^	−0.25^*^	—		
4. Experimental condition	0.52	0.50	0.17	0.26^*^	−0.27^*^^*^	—	
5. Psychological safety	4.66	1.05	0.19	−0.02	0.16	0.02	—
6. PCT	5.20	1.78	−0.01	0.23^*^	−0.14	0.25^*^	−0.04

*N* = 101. PCT, perceived contribution to team effectiveness.

^a^0 = Nurses, 1 = Physicians. ^b^0 = No voice, 1 = Voice.

* *p* < 0.05,

**p < 0.01.

To test hypothesis 1, which predicted that nurses who voice are more likely to be perceived as helpful for the team than those who do not voice, we computed an independent samples *t*-test. In line with prediction, findings revealed that nurses in the voice condition (*M* = 5.60, *SD* = 1.30) were seen as contributing more strongly to team effectiveness than nurses in the no-voice condition (*M* = 4.72, *SD* = 2.14), *t*(91) = −2.37, *p* = 0.020.

To test hypothesis 2, that is, the moderating role of psychological safety in the relationship between voice and endorsement, we computed a multiple regression analysis using the PROCESS tool for SPSS (Hayes, 2017). Note that we centered psychological safety to ease interpretation of the results. In line with best-practice recommendations (Bernerth and Aguinis, 2016), we tested our proposed interaction hypothesis with and without the inclusion of relevant control variables. Supporting our hypothesis, we found a significant interaction between the experimental condition and psychological safety on perceived contribution to team effectiveness in our model using covariates (Table 2; *B* = 0.86, *SE* = 0.38, *t* = 2.27*, p* = 0.026; Δ*R^2^* = 0.05). We also found a significant interaction between psychological safety and condition in our model without covariates (Table 3; *B* = 1.00, *SE* = 0.36, *t* = 2.77*, p* = 0.007; Δ*R^2^* = 0.08).

**Table 2:** Moderated multiple regression results predicting perceived contribution to team effectiveness (PCT; including control variables).

Variable	PCT		
	*B*	*SE*	*t*
*Control variables*			
Professional role^a^	−0.35	0.48	−0.73
Work experience	0.02	0.02	1.12
Gender	−0.19	0.43	−0.45
*Predictors*			
Experimental condition^b^	0.87^*^	0.39	2.2
Psychological safety	−0.57	0.3	−1.86
*Interaction effect*			
Exp. cond. × Psych. safety	0.86*	0.38	2.27

*N* = 93.

a 0 = Nurses, 1 = Physicians.

b 0 = No voice, 1 = Voice.

* *p* < 0.05.

**Table 3:** Moderated multiple regression results predicting perceived contribution to team effectiveness (PCT; without control variables).

	PCT		
	*B*	*SE*	*t*
*Main effects*			
Experimental condition^a^	0.94^*^	0.37	2.57
Psychological safety	−0.70^*^	0.29	−2.48
*Interaction effect*			
Exp. cond. × Psych. safety	1.00^**^	0.36	2.77

*N* = 93.

a 0 = No voice, 1 = Voice.

* *p* < 0.05;

** *p* < 0.01.

**Figure 1:**
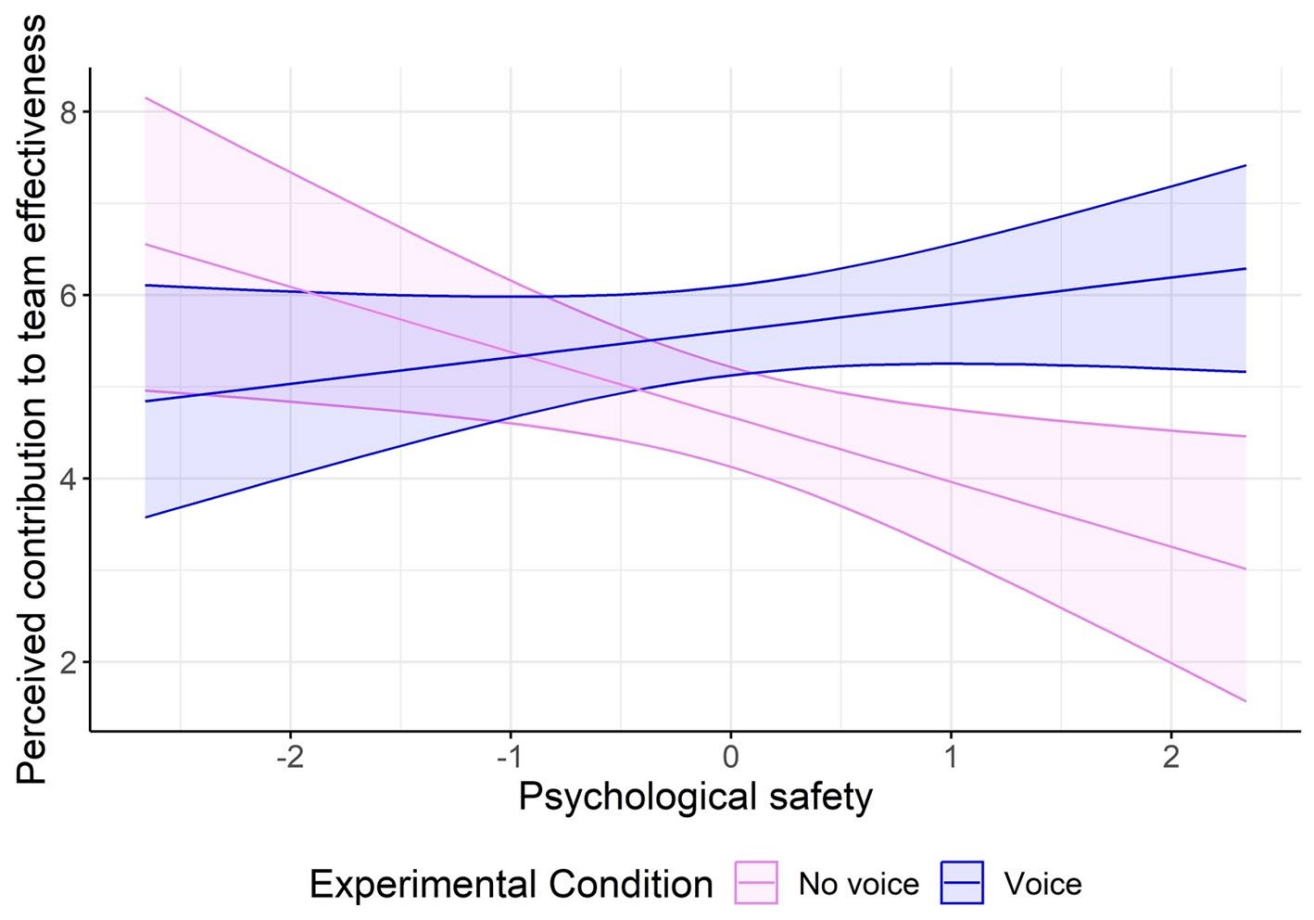
Interaction effect between experimental condition (no voice vs. voice) at different levels of psychological safety on perceived contribution to team effectiveness.

Figure 1 depicts the interaction effect and visualizes the slopes for voice versus no voice under different levels of psychological safety. Simple slope analyses indicated a significant effect at higher levels of psychological safety, showing that when psychological safety was perceived to be high (i.e., 1 SD above the mean), participants evaluated the nurse who voiced concerns with the treatment and spoke up with an alternative suggestion as more valuable for team decision-making than the nurse who did not voice concerns or offer alternative suggestions (*B* = 0.95, *SE* = 1.88, *t* = 3.76*, p* < 0.001). In contrast, when psychological safety was perceived to be low (i.e., 1 SD below the mean), there was no significant relationship between voice and perceived contribution to team effectiveness (*B* = −0.98, *SE* = −0.04, *t* = −0.07*, ns*).

As an additional analysis, we also analyzed our data using the subsample of physicians (*n* = 64) to better understand how physicians as higher-ranking individuals evaluate nurses who voice or remain silent with suggestions. Computing a moderated regression analysis, we also found a significant interaction between psychological safety and experimental condition on endorsement in this group (*n* = 64; *B* = 1.31, *SE* = 0.44, *t* = 2.98, *p* = 0.004). Simple slopes analyses revealed the same direction of effects as in our overall sample with physicians evaluating voicing nurses more positively at high (i.e., 1 SD above the mean; *B* = 2.48, *SE* = 0.59, *t* = 4.20, *p* < 0.001) but not at low (i.e., 1 SD below the mean) levels of psychological safety (*B* = 0.01, *SE* = 0.58, *t* = 0.03, *p* = 0.974). Consistent with our findings from the overall sample, nurses who voiced concerns were generally considered as more helpful for the team by physicians, but this effect tended to increase and decrease with increasing and decreasing levels of psychological safety. Finally, we also computed a three-way interaction between professional role, experimental condition, and psychological safety on perceived contribution to team effectiveness but found no significant three-way interaction effect (*B* = 0.54, *SE* = 0.98, *p* = 0.59). Overall, this suggests that participants’ function (i.e., being a nurse or a physician) did not significantly affect their judgment of the nurse.

## Discussion

This study shows that when healthcare team members feel psychologically safe, they evaluate nurses who speak up as contributing more positively to the team than those who do not voice their concerns. Recent research indicates that voice can have positive interpersonal consequences, including higher recognition by superiors, higher perceived social status or better leadership abilities (Howell et al., 2015; McClean et al., 2018; Weiss et al., 2018).

### Theoretical implications

Our study complements and extends research on consequences of voice by showing that psychological safety poses a boundary condition for evaluations of voice in functionally and status-diverse teams such as healthcare teams. In line with a social-cognitive perspective, people seem to “read the wind,” that is, they look for cues in their immediate work context to determine whether another person’s voice behavior may or may not be beneficial for the group (Fiske, 1993; Dutton et al., 1997). This seems particularly relevant when individuals assess the value of voice from a lower-ranking team member as such voice may challenge hierarchical boundaries and authority gradients (Magee and Galinsky, 2008).

We also complement research that has shown that group-or climate-related factors in teams such as psychological safety or group-voice climate are antecedents of voice (Detert and Trevino, 2010; Morrison et al., 2011). Our findings show that perceptions of psychological safety are also relevant for *evaluations of* voice. Nurses and physicians who feel psychologically safe consider nurses’ voice, versus withholding of voice, as more helpful to team decision-making. Even though we did not find perceptions of low psychological safety to significantly diminish the evaluation of voicers, we found that under this condition people do not seem to recognize the added value of voice coming from a nurse to potentially improve team outcomes. This resonates with the notion of psychological safety as an enabler for organizational learning and change (Schein, 1985; Edmondson and Lei, 2014). Because we consider reactions toward voicing, this naturally has implications for individuals’ further attempts to induce change at work. When leaders and coworkers believe that speaking up is helpful for the team (which this work shows is contingent on their own psychological safety), individuals who voice may continue to speak up versus learn that it is not advisable to do so.

As noted by Schein (1993), psychological safety reduces learning anxiety which is often prevalent when people are confronted with novel or contradictory information. When people feel that divergent opinions create ambiguity and uncertainty rather than an opportunity for reflection and adaptation, they do not perceive others who voice as helpful contributors to decision-making, and consequently, an opportunity for team reflection and learning is neglected. The fact that we found no differences between nurses and physicians in evaluating voice vs. withholding of voice from a nurse further underlines that psychological safety has implications regardless of employees’ formal role or status within an organization. Physicians were just as likely as nurses to be guided by their perceptions of psychological safety when evaluating a nurse who speaks up or remains silent. While most previous work has conceptualized psychological safety as a shared team-or organizational-level concept (e.g., Edmondson and Lei, 2014; Roussin et al., 2016), we highlight psychological safety as a social-cognitive variable that differs across individuals within the same work context. This extends our theoretical understanding of psychological safety, as the same organizational context may be perceived as a psychologically safe vs. unsafe context by different individuals. The variance in psychological safety that was evident in our sample (composed of individuals from the same work unit) suggests that individual experiences and sense-making processes likely shape different perceptions across individuals within the same organization, unit, or team.

Our results also have implications within the broader context of teamwork. Although functionally diverse teams may have more information available due to team members’ divergent expertise, they are often unable to reap those benefits. Due to social categorization processes and “us” vs. “them” distinctions, stereotyping, discrimination, and disparaging treatment of out-group members are often prevalent in diverse teams, with negative implications for team processes and outcomes (Hogg and Terry, 2000). Studies have shown that psychological safety moderates the extent to which diverse teams can leverage the benefits of their diverse demographic, functional, or cognitive backgrounds or when they have diverging viewpoints (e.g., Bradley et al., 2012; Kirkman et al., 2013; Martins et al., 2013; Jetten and Hornsey, 2014). For example, Martins et al. (2013) showed that high levels of psychological safety can buffer the negative effect of expertise diversity on team performance. The authors suggested that psychological safety fosters a more inclusive team climate in which the capabilities of different team members can be best utilized. We add to this line of research by showing that a high level of psychological safety is positively associated with an appreciation of those who constructively challenge the status quo to the benefit of the team.

### Limitations and future research

There are some limitations of our study that are worth noting. Although we were able to conduct a randomized between-subjects experiment with working healthcare professionals and systematically investigate responses to voice, participants were not actually immersed in the situation. This may raise concerns about participants responding in a socially desirable manner and evaluating the nurse’s voice behavior in a way that was overly positive. In actual teamwork or interpersonal episodes, factors beyond the content of the message such as timing or tone (Whiting et al., 2012) as well as liking and other interpersonal factors may also affect evaluations of voice. Thus, we encourage the use of laboratory studies (e.g., using medical or other teamwork simulations) where voice behavior can be manipulated *via* a confederate and subsequent evaluations can be observed and assessed more validly.

Second, our sample was slightly skewed, as we had more physicians participating in the study than nurses. However, we actually consider this a plus, as we thus had more individuals from the higher-ranking profession evaluating an individual from a lower-ranking profession. Studies investigating voice behavior in healthcare settings have shown that nurses are particularly hesitant to voice concerns to physicians because they feel that it is not their place to speak up and that they would bypass the hierarchy when doing so (Edmondson, 1996). Although our findings suggest that evaluations of voice may be more determined by perceptions of psychological safety rather than one’s professional role and hierarchical rank, future research should assess the impact of professional role on voice endorsement using larger samples from different team contexts.

Relatedly, our sample was drawn from a single department in a hospital, which may limit the generalizability to other organizational contexts. Because perceptions of psychological safety likely differ across occupational contexts and organizational levels, future research should examine other team and organizational contexts. It would also be valuable to adopt a multi-level approach to investigate how within-and between team differences in psychological safety affect evaluations of voice.

### Practical implications

One important practical implication that can be derived from our findings is that team leaders, which, in healthcare teams are represented by senior physicians, need to be mindful of the importance of psychological safety in affecting interpersonal evaluations and team processes. One way in which leaders may increase psychological safety is through communication with their subordinates. For example, a study investigating the effects of inclusive leader language within professionally diverse teams showed that leader attempts to foster voice behavior need to be adapted to different team members: team members from a lower status profession (i.e., nurses) required more affirmation that their voice is appreciated than team members from a higher status profession (i.e., physicians; Weiss et al., 2018). Thus, using collective language and explicitly inviting nurses to speak up can help build psychological safety in the team which not only increases voice but may also foster positive responses to it. Apart from inviting subordinates to speak up, leaders should also foster positive responses to voice within a team. Recent research shows that team leaders who openly seek and discuss feedback can build a climate of trust and psychological safety (Coutifaris and Grant, 2022). This signals to employees that their input is welcome and may result in sustained efforts to contribute to the team.

A further practical implication is that healthcare teams may also reflect their perceptions of psychological safety in the context of after-event reviews, that is, short debriefings that happen after a specific teamwork episode and that are frequently adopted in high-risk contexts (e.g., Ellis and Davidi, 2005). Such after event-reviews have been shown to decrease the perception of hierarchical barriers and increase voice behavior in teams (Weiss et al., 2017).

## Conclusion

Emerging research focuses on understanding how individuals who voice suggestions or work-related concerns are evaluated by others. We highlight psychological safety as an important moderator in healthcare teams that affects whether nurses who voice critical input are seen as more valuable contributors for team decision-making than those who withhold their concerns. Our findings underline that individuals rely on cues from the environment when determining the value of voice.

## Data availability statement

The raw data supporting the conclusions of this article will be made available by the authors upon request, without undue reservation.

## Ethics statement

The studies involving human participants were reviewed and approved by Institutional Review Board NYU Langone. The patients/participants provided their written informed consent to participate in this study.

## Author contributions

MW developed the research question, designed the study, performed the statistical analyses, and drafted the manuscript. EM supported the theoretical development of the manuscript. DS provided access to the sample and helped with the design of the experimental manipulations. All authors contributed to the article and approved the submitted version.

## Funding

This research has partly been funded by a grant from the Swiss National Science Foundation (Grant No. P2EZP1_159082) awarded to MW.

## Conflict of interest

The authors declare that the research was conducted in the absence of any commercial or financial relationships that could be construed as a potential conflict of interest.

## Publisher’s note

All claims expressed in this article are solely those of the authors and do not necessarily represent those of their affiliated organizations, or those of the publisher, the editors and the reviewers. Any product that may be evaluated in this article, or claim that may be made by its manufacturer, is not guaranteed or endorsed by the publisher.

## Footnotes

^1^We note that the reported results are consistent when including participants who failed the manipulation check.

## References

[ref1] Akşin Z. Deo S. Jónasson J. O. Ramdas K. (2021). Learning from many: partner exposure and team familiarity in fluid teams. Manag. Sci. 67, 854–874. doi: 10.1287/mnsc.2019.3576

[ref2] Apker J. Propp K. M. Zabava Ford W. S. (2005). Negotiating status and identity tensions in healthcare team interactions: an exploration of nurse role dialectics. J. Appl. Commun. Res. 33, 93–115. doi: 10.1080/00909880500044620

[ref3] Argyris C. (1993). Knowledge for Action: A Guide to Overcoming Barriers to Organizational Change. San Francisco, CA: Jossey-Bass

[ref4] Avgerinos E. Fragkos I. Huang Y. (2020). Team familiarity in cardiac surgery operations: the effects of hierarchy and failure on team productivity. Hum. Relat. 73, 1278–1307. doi: 10.1177/0018726719857122

[ref5] Baer M. Frese M. (2003). Innovation is not enough: climates for initiative and psychological safety, process innovations, and firm performance. J. Organ. Behav. 24, 45–68. doi: 10.1002/job.179

[ref6] Bernerth J. B. Aguinis H. (2016). A critical review and best-practice recommendations for control variable usage. Pers. Psychol. 69, 229–283. doi: 10.1111/peps.12103

[ref7] Bradley B. H. Postlethwaite B. E. Klotz A. C. Hamdani M. R. Brown K. G. (2012). Reaping the benefits of task conflict in teams: the critical role of team psychological safety climate. J. Appl. Psychol. 97, 151–158. doi: 10.1037/a0024200, PMID: 21728397

[ref8] Burris E. R. (2012). The risks and rewards of speaking up: managerial responses to employee voice. Acad. Manag. J. 55, 851–875. doi: 10.5465/amj.2010.0562

[ref9] Burris E. R. Rockmann K. W. Kimmons Y. S. (2017). The value of voice to managers: employee identification and the content of voice. Acad. Manag. J. 60, 2099–2125. doi: 10.5465/amj.2014.0320

[ref10] Carmeli A. Gittell J. H. (2009). High-quality relationships, psychological safety, and learning from failures in work organizations. J. Organ. Behav. 30, 709–729. doi: 10.1002/job.565

[ref11] Castelao E. F. Russo S. G. Riethmüller M. Boos M. (2013). Effects of team coordination during cardiopulmonary resuscitation: a systematic review of the literature. J. Crit. Care 28, 504–521. doi: 10.1016/j.jcrc.2013.01.005, PMID: 23602030

[ref12] Cataldo C. G. Raelin J. D. Lambert M. (2009). Reinvigorating the struggling organization: the unification of Schein’s oeuvre into a diagnostic model. J. Appl. Behav. Sci. 45, 122–140. doi: 10.1177/0021886308328849

[ref13] Chamberlin M. Newton D. W. Lepine J. A. (2017). A meta-analysis of voice and its promotive and prohibitive forms: identification of key associations, distinctions, and future research directions. Pers. Psychol. 70, 11–71. doi: 10.1111/peps.12185

[ref14] Cott C. (1997). “We decide, you carry it out”: a social network analysis of multidisciplinary long-term care teams. Soc. Sci. Med. 45, 1411–1421. doi: 10.1016/S0277-9536(97)00066-X, PMID: 9351158

[ref15] Coutifaris C. G. Grant A. M. (2022). Taking your team behind the curtain: the effects of leader feedback-sharing and feedback-seeking on team psychological safety. Organ. Sci. 33, 1574–1598. doi: 10.1287/orsc.2021.1498

[ref16] Detert J. R. Burris E. R. (2007). Leadership behavior and employee voice: is the door really open? Acad. Manag. J. 50, 869–884. doi: 10.5465/amj.2007.26279183

[ref17] Detert J. R. Burris E. R. Harrison D. A. Martin S. R. (2013). Voice flows to and around leaders: understanding when units are helped or hurt by employee voice. Adm. Sci. Q. 58, 624–668. doi: 10.1177/0001839213510151

[ref18] Detert J. R. Trevino L. K. (2010). Speaking up to higher-ups: how supervisors and skip-level leaders influence employee voice. Organ. Sci. 21, 249–270. doi: 10.1287/orsc.1080.0405

[ref19] Dunford J. V. Davis D. P. Ochs M. Doney M. Hoyt D. B. (2003). Incidence of transient hypoxia and pulse rate reactivity during paramedic rapid sequence intubation. Ann. Emerg. Med. 42, 721–728. doi: 10.1016/S0196-0644(03)00660-7, PMID: 14634593

[ref20] Dutton J. E. Ashford S. J. Oneill R. M. Hayes E. Wierba E. E. (1997). Reading the wind: how middle managers assess the context for selling issues to top managers. Strateg. Manag. J. 18, 407–423. doi: 10.1002/(SICI)1097-0266(199705)18:5<407::AID-SMJ881>3.0.CO;2-J

[ref21] Edmondson A. C. (1996). Learning from mistakes is easier said than done: group and organizational influences on the detection and correction of human error. J. Appl. Behav. Sci. 32, 5–28. doi: 10.1177/0021886304263849

[ref22] Edmondson A. C. (1999). Psychological safety and learning behavior in work teams. Adm. Sci. Q. 44, 350–383. doi: 10.2307/2666999

[ref100] Edmondson A. C. Bohmer R. M. Pisano G. P. (2001). Disrupted routines: Team learning and new technology implementation in hospitals. Administr. Sci. Quart. 46, 685–716., PMID: 30407041

[ref23] Edmondson A. C. Lei Z. (2014). Psychological safety: the history, renaissance, and future of an interpersonal construct. Annu. Rev. Organ. Psych. Organ. Behav. 1, 23–43. doi: 10.1146/annurev-orgpsych-031413-091305

[ref24] Ellis S. Davidi I. (2005). After-event reviews: drawing lessons from successful and failed experience. J. Appl. Psychol. 90, 857–871. doi: 10.1037/0021-9010.90.5.857, PMID: 16162059

[ref25] Fiske S. T. (1993). Social cognition and social perception. Annu. Rev. Psychol. 44, 155–194. doi: 10.1146/annurev.ps.44.020193.001103 8434891

[ref26] Hautz W. E. Kämmer J. E. Schauber S. K. Spies C. D. Gaissmaier W. (2015). Diagnostic performance by medical students working individually or in teams. JAMA 313, 303–304. doi: 10.1001/jama.2014.15770 25603003

[ref27] Hayes A. F. (2017). Introduction to Mediation, Moderation, and Conditional Process Analysis: A Regression-Based Approach. New York, NY: Guilford Press.

[ref28] Hogg M. Terry D. J. (2000). Social identity and self-categorization processes in organizational contexts. Acad. Manag. Rev. 25, 121–140. doi: 10.5465/AMR.2000.2791606

[ref29] Howell T. M. Harrison D. A. Burris E. R. Detert J. R. (2015). Who gets credit for input? Demographic and structural status cues in voice recognition. J. Appl. Psychol. 100, 1765–1784. doi: 10.1037/apl0000025, PMID: 25915784

[ref30] Hunziker S. Johansson A. C. Tschan F. Semmer N. K. Rock L. Howell M. D.. (2011). Teamwork and leadership in cardiopulmonary resuscitation. J. Am. Coll. Cardiol. 57, 2381–2388. doi: 10.1016/j.jacc.2011.03.017 21658557

[ref31] Jetten J. Hornsey M. J. (2014). Deviance and dissent in groups. Annu. Rev. Psychol. 65, 461–485. doi: 10.1146/annurev-psych-010213-115151, PMID: 23751035

[ref32] Kahn W. A. (1990). Psychological conditions of personal engagement and disengagement at work. Acad. Manag. J. 33, 692–724. doi: 10.5465/256287

[ref33] Kobayashi H. Pian-Smith M. Sato M. Sawa R. Takeshita T. Raemer D. (2006). A cross-cultural survey of residents’ perceived barriers in questioning/challenging authority. BMJ Qual. Saf. 15, 277–283. doi: 10.1136/qshc.2005.017368, PMID: 16885253 PMC2564023

[ref200] Kirkman B. L. Cordery J. L. Mathieu J. Rosen B. Kukenberger M. (2013). Global organizational communities of practice: The effects of nationality diversity, psychological safety, and media richness on community performance. Human Relations 66, 333–362.

[ref35] Koo T. K. Li M. Y. (2016). A guideline of selecting and reporting intraclass correlation coefficients for reliability research. J. Chiropr. Med. 15, 155–163. doi: 10.1016/j.jcm.2016.02.012, PMID: 27330520 PMC4913118

[ref400] Krenz H. Burtscher M. J. Grande B. Kolbe M. (2020). Nurses’ voice: the role of hierarchy and leadership. Leadership in Health Services 33, 12–26.

[ref36] Lam C. F. Lee C. Sui Y. (2019). Say it as it is: consequences of voice directness, voice politeness, and voicer credibility on voice endorsement. J. Appl. Psychol. 104, 642–658. doi: 10.1037/apl0000358, PMID: 30407041

[ref39] Magee J. C. Galinsky A. D. (2008). 8 social hierarchy: the self-reinforcing nature of power and status. Acad. Manag. Ann. 2, 351–398. doi: 10.5465/19416520802211628

[ref500] Martins L. L. Schilpzand M. C. Kirkman B. L. Ivanaj S. Ivanaj V. (2013). A contingency view of the effects of cognitive diversity on team performance: The moderating roles of team psychological safety and relationship conflict. Small Group Research. 44, 96–126.

[ref40] Maynes T. D. Podsakoff P. M. (2014). Speaking more broadly: an examination of the nature, antecedents, and consequences of an expanded set of employee voice behaviors. J. Appl. Psychol. 99, 87–112. doi: 10.1037/a0034284, PMID: 24041119

[ref41] McClean E. J. Martin S. R. Emich K. J. Woodruff C. T. (2018). The social consequences of voice: an examination of voice type and gender on status and subsequent leader emergence. Acad. Manag. J. 61, 1869–1891. doi: 10.5465/amj.2016.0148

[ref600] Mesmer-Magnus J. R. DeChurch L. A. (2009). Information sharing and team performance: a meta-analysis. J. Appl. Psychol. 94, 535–546. 19271807 10.1037/a0013773

[ref42] Morrison E. W. (2014). Employee voice and silence. Annu. Rev. Organ. Psych. Organ. Behav. 1, 173–197. doi: 10.1146/annurev-orgpsych-031413-091328

[ref43] Morrison E. W. (2023). Employee voice and silence: taking stock a decade later. Annu. Rev. Organ. Psych. Organ. Behav. 10, 79–107. doi: 10.1146/annurev-orgpsych-120920-054654

[ref44] Morrison E. W. Milliken F. J. (2000). Organizational silence: a barrier to change and development in a pluralistic world. Acad. Manag. Rev. 25, 706–725. doi: 10.5465/amr.2000.3707697

[ref45] Morrison E. W. Wheeler-Smith S. L. Kamdar D. (2011). Speaking up in groups: a cross-level study of group voice climate and voice. J. Appl. Psychol. 96, 183–191. doi: 10.1037/a0020744, PMID: 20718517

[ref46] Nembhard I. M. Edmondson A. C. (2006). Making it safe: the effects of leader inclusiveness and professional status on psychological safety and improvement efforts in health care teams. J. Organ. Behav. 27, 941–966. doi: 10.1002/job

[ref47] Nijstad B. A. Berger-Selman F. De Dreu C. K. W. (2014). Innovation in top management teams: minority dissent, transformational leadership, and radical innovations. Eur. J. Work Organ. Psychol. 23, 310–322. doi: 10.1080/1359432X.2012.734038

[ref48] O’Donovan R. De Brún A. McAuliffe E. (2021). Healthcare professionals experience of psychological safety, voice, and silence. Front. Psychol. 12:626689. doi: 10.3389/fpsyg.2021.626689, PMID: 33679547 PMC7933795

[ref49] O’Driscoll B. R. Howard L. S. Earis J. Mak V. (2017). British Thoracic Society guideline for oxygen use in adults in healthcare and emergency settings. BMJ Open Respir. Res. 4:e000170. doi: 10.1136/bmjresp-2016-000170, PMID: 28883921 PMC5531304

[ref50] Roussin C. J. MacLean T. L. Rudolph J. W. (2016). The safety in unsafe teams: a multilevel approach to team psychological safety. J. Manag. 42, 1409–1433. doi: 10.1177/0149206314525204

[ref51] Russo M. Buonocore F. Ferrara M. (2015). Motivational mechanisms influencing error reporting among nurses. J. Manag. Psychol. 30, 118–132. doi: 10.1108/JMP-02-2013-0060

[ref300] Schein E. H. (1985). Organizational culture and leadership. San Francisco: Jossey-Bass.

[ref53] Schein E. H. (1993). On dialogue, culture, and organizational learning. Organ. Dyn. 22, 40–51. doi: 10.1016/0090-2616(93)90052-3

[ref54] Schmutz J. Manser T. (2013). Do team processes really have an effect on clinical performance? A systematic literature review. Br. J. Anaesth. 110, 529–544. doi: 10.1093/bja/aes513 23454826

[ref56] Steinmetz J. Xu Q. Fishbach A. Zhang Y. (2016). Being observed magnifies action. J. Pers. Soc. Psychol. 111, 852–865. doi: 10.1037/pspi0000065 27454927

[ref57] Sundstrom E. De Meuse K. P. Futrell D. (1990). Work teams: applications and effectiveness. Am. Psychol. 45, 120–133. doi: 10.1037/0003-066X.45.2.120

[ref58] Uitdewilligen S. Waller M. J. (2018). Information sharing and decision-making in multidisciplinary crisis management teams. J. Organ. Behav. 39, 731–748. doi: 10.1002/job.2301

[ref59] Waller B. M. Hope L. Burrowes N. Morrison E. R. (2011). Twelve (not so) angry men: managing conversational group size increases perceived contribution by decision makers. Group Process. Intergroup Relat. 14, 835–843. doi: 10.1177/1368430211407099

[ref60] Weiss M. Kolbe M. Grote G. Dambach M. Marty A. Spahn D. R.. (2014). Agency and communion predict speaking up in acute care teams. Small Group Res. 45, 290–313. doi: 10.1177/1046496414531495

[ref61] Weiss M. Kolbe M. Grote G. Spahn D. R. Grande B. (2017). Why didn’t you say something? Effects of after-event reviews on voice behaviour and hierarchy beliefs in multi-professional action teams. Eur. J. Work Organ. Psychol. 26, 66–80. doi: 10.1080/1359432X.2016.1208652

[ref62] Weiss M. Kolbe M. Grote G. Spahn D. R. Grande B. (2018). We can do it! Inclusive leader language promotes voice behavior in multi-professional teams. Leadersh. Q. 29, 389–402. doi: 10.1016/j.leaqua.2017.09.002

[ref63] Weiss M. Morrison E. W. (2019). Speaking up and moving up: how voice can enhance employees’ social status. J. Organ. Behav. 40, 5–19. doi: 10.1002/job.2262

[ref64] Whiting S. W. Maynes T. D. Podsakoff N. P. Podsakoff P. M. (2012). Effects of message, source, and context on evaluations of employee voice behavior. J. Appl. Psychol. 97, 159–182. doi: 10.1037/a0024871, PMID: 21842973

