# Supplementary material for: I like what you are saying, but only if i feel safe: Psychological safety moderates the relationship between voice and perceived contribution to healthcare team effectiveness
Source: Front Psychol. 2023 Apr 17;14:1129359. doi: 10.3389/fpsyg.2023.1129359 (PMC10150701; doi:10.3389/fpsyg.2023.1129359)
Supplement: Supplementary file 1 [file Table_1.DOCX]

**Appendix A**

1. **Voice condition for physicians
   Please imagine you are in the following situation:**

You are in the ER together with a nurse, a resident and an attending emergency physician. You are examining a 25-year old male patient who was involved in a severe car accident and arrived 10 minutes ago. The patient suffers from multiple injuries such as a fracture of the left femur, rib fractures, and mild-moderate traumatic brain injury (symptoms point to a concussion). According to initial assessment of the admitting emergency resident he has a GCS of 12.

Because of the various injuries and the state of the patient, you feel that it is necessary to check for additional internal injuries and to perform a CT.

One of the nurses suggests to intubate the patient before he is transferred to radiology. She explains to you that she is concerned that the patient might get unstable during the CT and feels that it is safer to secure the airway beforehand (current oxygen saturation is 90%).

1. **No voice condition for physicians**

**Please imagine you are in the following situation:**

You are in the ER together with a nurse, a resident and an attending emergency physician. You are examining a 25-year old male patient who was involved in a severe car accident and arrived 10 minutes ago. The patient suffers from multiple injuries such as a fracture of the left femur, rib fractures, and mild-moderate traumatic brain injury (symptoms point to a concussion). According to initial assessment of the admitting emergency resident he has a GCS of 12.

Because of the various injuries and the state of the patient, you feel that it is necessary to check for additional internal injuries and to perform a CT.

One of the nurses thinks that it’s important to intubate the patient before he is transferred to radiology. She is concerned that the patient might get unstable during the CT and feels that it is safer to secure the airway beforehand (current oxygen saturation is 90%). However, she decides not to bring up these concerns to you.

1. **Voice condition for nurses**

**Please imagine you are in the following situation:**

You are in the ER together with another nurse, a resident and an attending emergency physician. You are examining a 25-year old male patient who was involved in a severe car accident and arrived 10 minutes ago. The patient suffers from multiple injuries such as a fracture of the left femur, rib fractures, and mild-moderate traumatic brain injury (symptoms point to a concussion). According to initial assessment of the admitting emergency resident he has a GCS of 12.

Because of the various injuries and the state of the patient, the resident and the attending emergency physician feel that it is necessary to check for additional internal injuries and to perform a CT.

The other nurse suggests to intubate the patient before he is transferred to radiology. She explains to the physicians that she is concerned that the patient might get unstable during the CT and feels that it is safer to secure the airway beforehand (current oxygen saturation is 90%).

1. **No voice condition for nurses**

**Please imagine you are in the following situation:**

You are in the ER together with another nurse, a resident and an attending emergency physician. You are examining a 25-year old male patient who was involved in a severe car accident and arrived 10 minutes ago. The patient suffers from multiple injuries such as a fracture of the left femur, rib fractures, and mild-moderate traumatic brain injury (symptoms point to a concussion). According to initial assessment of the admitting emergency resident he has a GCS of 12.

Because of the various injuries and the state of the patient, the resident and the attending emergency physician feel that it is necessary to check for additional internal injuries and to perform a CT.

The other nurse feels that it’s important to intubate the patient before he is transferred to radiology. She is concerned that the patient might get unstable during the CT and feels that it is safer to secure the airway beforehand (current oxygen saturation is 90%). However, she decides not to bring up these concerns to the physicians.
